# *In vitro* gastrointestinal digestion study of two wheat cultivars and evaluation of xylanase supplementation

**DOI:** 10.1186/s40104-015-0002-7

**Published:** 2015-02-15

**Authors:** Mickael Lafond, Bernard Bouza, Sandrine Eyrichine, Friedrich Rouffineau, Luc Saulnier, Thierry Giardina, Estelle Bonnin, Aurélie Preynat

**Affiliations:** iSm2 - BiosCiences UMR 7313, Aix Marseille Université, Centrale Marseille, CNRS, Marseille, France; Adisseo France S.A.S., Centre d’Expertise et de Recherche en Nutrition, Commentry, France; INRA, UR 1268 - Biopolymères - Interactions – Assemblages, Nantes, France

**Keywords:** *in vitro* TNO gastrointestinal model-1, *Talaromyces versatilis*, Wheat cultivars, XynB, XynD

## Abstract

**Background:**

The filamentous fungus *Talaromyces versatilis* is known to improve the metabolizable energy of wheat-based poultry diets thanks to its ability to produce a pool of CAZymes and particularly endo-β(1,4)-xylanases. In order to appreciate their *in vivo* mode of action, the supplementation effect of two of its xylanases, XynD and XynB from families GH10 and GH11 respectively, have been evaluated on two different wheat cultivars Caphorn and Isengrain, which were chosen amongst 6 varieties for their difference in non starch polysaccharides content and arabinoxylan composition.

**Results:**

Polysaccharides digestion was followed during 6 h along the digestive tract using the TNO gastrointestinal model-1, to mimic monogastric metabolism. Polysaccharide degradation appeared to occur mainly at the jejunal level and was higher with Isengrain than with Caphorn. For both cultivars, XynD and XynB supplementation increased notably the amount of reducing end sugars into the jejuno-ileal dialysates, which has been confirmed by a valuable increase of the soluble glucose into the jejunal dialysates.

**Conclusions:**

The amounts of arabinose and xylose into the dialysates and ileal deliveries increased consequently mainly for Caphorn, suggesting that XynD and XynB supplementation in wheat-based diet could alleviate the anti-nutritional effects of arabinoxylans by limiting the physical entrapment of starch and could increase the available metabolizable energy.

## Background

Due to its high content in storage polysaccharides (*i.e.* starch), wheat is a crucial source of energy in poultry diets contributing up to 650 g/kg diet for finishing broilers [1,2]. However, wheat grain contains 12-18% non-starch polysaccharides (NSP), which may result in low performance of growing broilers [3]. In wheat, as in other cereals, the physical entrapment of starch and protein by cell wall polysaccharides is one of the possible mechanisms by which NSP exert an anti-nutritional effect [4-6]. Anti-nutritional effects of NSP in many cereals are also due to the presence of high molecular weight soluble polysaccharides that enhance the viscosity in the digestive tract and reduce nutrient absorption. Enzymatic degradation of both soluble and insoluble NSP can also favour more effective hindgut fermentation and thereby improves overall energy utilization [7]. Arabinoxylans (AXs), the main cell wall polysaccharides in wheat, are composed predominantly of two pentoses, arabinose and xylose [8]. These polysaccharides are formed from a linear backbone of β(1,4)-linked D-xylose on which α-L-arabinofuranosyl units are attached as single side-chain units through O2 and/or O3 [9]. Other constituents such as galactose and glucuronic acid may also be present as side chains in addition to arabinose in heteroxylan isolated from the outer tissues of the grain. Low amounts of ferulic acid are esterified to arabinose side-chains [10]. Several studies have shown that supplementation of NSP-degrading enzymes could alleviate the anti-nutritional effect of cell wall polysaccharides, notably by partially breaking down the AX [9,11-14].

The filamentous fungus *Penicillium funiculosum,* recently renamed *Talaromyces versatilis* according to the ICBN [15,16], produces a wide range of glycoside hydrolases (GH) (www.cazy.org; [17]). Recently, *T. versatilis* was shown to produce a large variety of key enzymes of interest for biomass deconstruction as the proteomic analysis of its secretome allowed the identification of 34 Carbohydrate-Active ENzymes (CAZymes) [18]. In previous studies, we have heterologously expressed (in *Pichia pastoris*) and characterized one endo-β(1,4)-xylanase from GH10 (XynD) [19] and four endo-β(1,4)-xylanases from GH11 (XynB, XynC, XynE and XynF) [20]. Despite the high diversity of xylanases in *T. versatilis* and the high complementarity of their enzyme activities and specificities [19,20], it appears that XynD and XynB present a particular interest for biotechnological applications even if their effect *in vivo* remains unknown to date.

As it is almost impossible to precisely assess the molecular effect of CAZymes along the animal’s guts, the *in vitro* TNO gastrointestinal model-1 (TIM-1) represents an alternate choice to improve our knowledge of the enzyme action along the digestive tract [21]. Indeed, TIM-1 model simulates the successive dynamic conditions in the gastric/small intestinal tract such as body temperature, pH, concentrations of bile salts and gastro-duodenal enzymes secreted in the successive subdivisions of the digestive tract. It also makes it possible to follow the kinetics of chyme transit through the stomach and proximal intestine and it simulates the absorption of low-molecular-weight molecules and water [22]. Previous studies on the evaluation of β-glucan and resistant starch digestibility using the TIM-1 method have shown similar results to that obtained *in vivo* (i.e. ileostomy patients) [23,24]. In addition, by using this *in vitro* model and AX extracted from different wheat cultivars, it has been shown that the AX structure might influence the improvement of digestibility related to supplementation with CAZymes [14,25]. However, the relationships between wheat characteristics, such as their NSP content and structure, and their responses to enzyme addition are not yet fully known and understood, especially in the gut. Moreover, the relationship between the *in vitro versus in vivo* modes of action of the enzymes is currently unknown.

The aim of the present study was to evaluate the effects of XynD and XynB on wheat digestion, with emphasis on the carbohydrate soluble fractions that are associated with major detrimental effects of NSP on animal performances [26]. In order to understand the relationship between the wheat characteristics and the wheat response to supplementation by both xylanases, two wheat cultivars (Caphorn and Isengrain) with differing carbohydrate composition and viscosity [27-29] were chosen among 6 cultivars to be compared. The TIM-1 model allowed the study of the polysaccharide digestion kinetics in the different compartments of the gastro-intestinal artificial tract by following the formed products.

## Methods

### Wheat cultivars

The wheat cultivars Caphorn, Tapidor, Apache, Oratorio, Aztec and Isengrain were harvested in 2008 and kindly provided by Euronutrition (St Symphorien, France).

### Enzymes

The recombinant XynD [GenBank: AJ634957.1] and XynB [GenBank: AJ489605.1] from the filamentous fungus *T. versatilis* were heterologously expressed in the methylotrophic yeast *Pichia pastoris* X33 and GS115, respectively. In both cases the protein was expressed under the methanol inducible promoter (*AOX1*). The culture supernatants were recovered by centrifugation (10 min, 4,000 *g*) after 3 days methanol induction and were concentrated using Ultracel^TM^ ultrafiltration membrane (3 kDa molecular weight cut-off, Poly-Ether-Sulfone, 4 bars, Millipore, Molsheim, France). The proteins were then submitted to a single purification step of gel filtration on a Sephacryl S200 column (GE Healthcare Europe GmbH, Velizy-Villacoublay, France), as previously described in Lafond *et al.* [19]. The trypsin from bovine pancreas, pepsin from porcine gastric mucosa and porcine bile extract were purchased from Sigma (St-Quentin-Fallavier, France). *Rhizopus* lipase was purchased from Amano Enzyme Europe (Oxfordshire, UK), and the pancreatin solution (that contain notably α(1,4)-amylase from pork), was from Paines & Byrne (Greenford, Middlesex, UK).

### Digestive solutions

Three digestive solutions were used in the TIM-1. The first solution used in the gastric compartment, was the gastric salt solution, which contains 1 g/L of sodium chloride, 1.1 g/L of potassium chloride and 0.15 g/L of calcium chloride di-hydrate. The second solution used in the duodenal compartment, is the duodenal salt solution composed of 2% of trypsin solution (2 mg/mL), 25% of pancreatin solution (7% w/w), 49% of bile extract solution and 25% of small-intestinal salt solution. The third solution is the small-intestinal salt solution, used in the duodenal, the jejunal and the ileal compartments, and contains 5 g/L of sodium chloride, 0.6 g/L of potassium chloride and 0.3 g/L of calcium chloride di-hydrate.

### Digestion conditions

The digestion medium for the TIM-1 trials was adapted from a previous study [14], and it was composed of 45 g (dry basis) ground wheat (grinding screen size 3 mm), 85 g of gastric salt solution, 5 g pepsin and lipase solution (90,000 and 11,200 U/mg, respectively). One unit of porcine pepsin corresponds to an increase of absorbance (at 280 nm) of 0.001 unit per min measured at 37°C and pH 2.0 using hemoglobin as substrate. One unit of pancreatic lipase is required to catalyze the formation of 1.0 μmol fatty acid from olive oil triacylglyceride in 1 h at 37°C and pH 7.7. Water was added to the gastric medium to adjust gastric content weight to 310 g. When added, XynD and XynB were used at 0.13 μL/g wheat, corresponding to 100.7 xylanase visco-unit/g wheat. One visco-unit of xylanase is defined as the amount of xylanase that hydrolyses low viscosity wheat AX (Megazyme International, Wicklow, Ireland), reducing its viscosity to change the relative fluidity of 1 (dimension less unit)/min under the assay conditions (pH 5.5 at 30°C).

### Digestion assays of wheat in TIM-1

**Figure 1:**
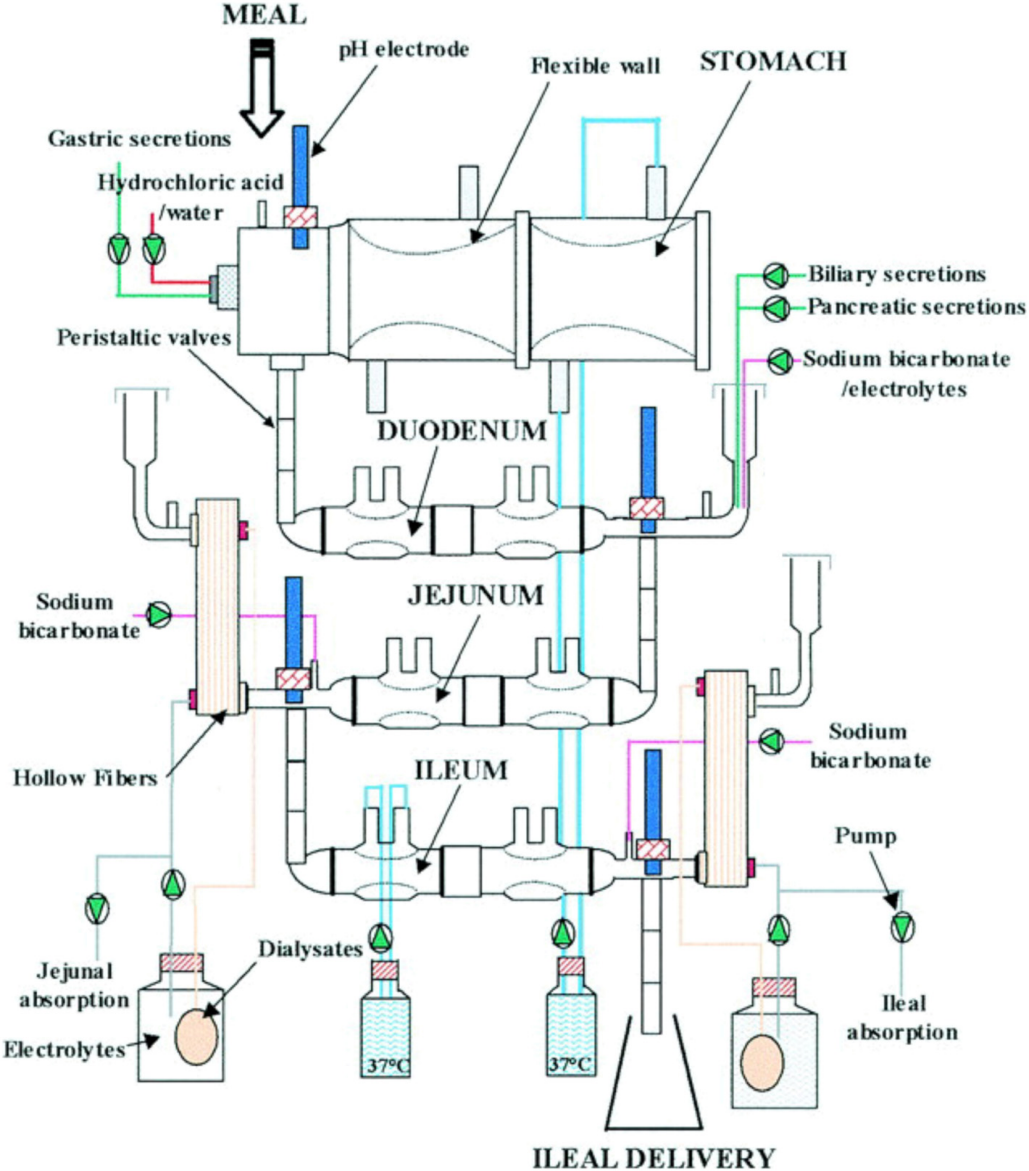
**Schematic diagram of the gastrointestinal** 1 **digestion model (TIM-1).**

The digestion assays were carried out on the TIM-1 developed at the TNO (Nederlandse Organisatie voor Toegepast Natuurwetenschappelijk Onderzoek) [21] (Figure 1). This *in vitro* model allows control of digestive secretions, pH, temperature and endogenous enzymes and mimics the gut peristalsis. The protocol of Minekus, adapted for pigs, was used along with some modifications to mimic poultry digestion [21]. The pH for the duodenal, jejunal and ileal compartments were adjusted at 6.5, 6.8 and 7.2, respectively. Before starting the incubation, the duodenal compartment was flushed with approximately 61 g of the duodenal salt solution described previously. The jejunal and ileal compartments were filled with 130 mL of the small-intestinal salt solution. The intestinal absorption was mimicked using haemodialyser HG-400 membranes with a molecular weight cut-off range of 5–10 kDa (Hospal Cobe, Lyon, France). The dialysis fluid was pumped at 10 mL/min and collected in the intervals 0–60, 60–120, 120–180, 180–240, and 240–360 min. The ileal effluents were collected between 0–120, 120–180, 180–240, and 240–360 min.

The TIM-1 model was fed with 45 g of Caphorn or Isengrain ground (3 mm) wheat grains and the effects of XynD and XynB were evaluated in duplicate with a 2 × 2 factorial arrangement.

### Samples recovery

Five types of sample were collected: ileal dialysate, jejunal dialysate, ileal deliveries, gastro-duodenal and jejuno-ileal residues. The ileal and jejunal dialysates were collected all along the digestion (see above) and were weighed and stored at −20°C until analysis. The ileal deliveries were also collected all along the digestion. All samples were centrifuged at 14,000 *g* for 10 min, supernatant and pellet were stored separately at −20°C. The gastro-duodenal residues and the jejuno-ileal residues correspond to the remaining fractions at the end of the digestion time-courses. Consequently, they were collected at the end of the run (*i.e.* 360 min) before to be centrifuged at 14,000 *g* for 10 min. The supernatants obtained, constituting the soluble fractions of the residues, were weighted and stored at −20°C until analysis whereas the pellets (insoluble part) were not taken into account for analysis.

### Sample analysis

The supernatants collected in the different compartments were analyzed for their content of reducing ends and carbohydrate compositions (monosaccharides and oligosaccharides). The released reducing ends were determined according to the dinitrosalicylic acid (DNS) method [30], using 96-well microplates and a KRL microplate-spectrophotometer (Kirial International, Couternon, France). The blank contained water instead of the sample. The data were expressed as absorbance units, since the extinction coefficients varied greatly depending on the nature of the reducing residue (data not shown). The sugar quantifications were performed in triplicate.

The monosaccharide composition of the wheat cultivars (Caphorn and Isengrain) and of the five samples recovered from the TIM-1 experiments (gastro-duodenal residues, jejuno-ileal residues, ileal deliveries, jejunal dialysis and ileal dialysis) was determined as alditol acetates by Gas Chromatography after acid hydrolysis [31]. The soluble extracts from wheat were prepared from 1 g of ground wheat grain in 4 mL of water [32]. The liquid fractions were hydrolyzed with H_2_SO_4_ (4 mol/L) in the presence of inositol as the internal standard (5 g/L) for 2 h at 100°C, whereas the pellets (providing from the gastro-duodenal and jejuno-ileal residues) were first pre-hydrolyzed with H_2_SO_4_ (13 mol/L) for 30 min at 25°C before inositol was added and hydrolysis was performed as for liquid fractions. After reduction sodium borohydride and acetylation, the samples were injected in the Gas–liquid Chromatography (GLC) system (Perkin-Elmer Autosystem, Courtaboeuf, France) using a 25 mm × 0.32 mm silica column (BP-225; J & W Scientific, Folsorn, CA, USA; temperature 205°C, carrier gas H_2_) and a flame ionisation detector. The results are means of duplicate and the variation coefficient was always less than 4%. The AX content was calculated as the sum of arabinose and xylose.

### Statistical analysis

Statistical analysis of repeated-measures data was performed using the mixed procedure of SAS 9.1.3 (SAS Institute, Inc., Cary, NC, USA). As the values were cumulated over time, the covariance structure was specified as ‘Auto regressive type 1’. The restricted maximum-likehood method was used to estimate covariance parameters, and the tests of fixed effects (in model, contrasts and least-square means) were performed using the residual degrees of freedom (df; using the ‘ddfm = RESIDUAL’ option). Time was specified as the factor of repeated-measures ANOVA determinations. Focusing on each compartment (jejunum and ileum) and their sum, the following multifactor statistical model was used to the enzyme effects on the polysaccharide digestion parameters: β π α η δ γ.

Y = α + β_i_ × wheat + γ_j_ × treat + d_k_ × Exp + π_l_ × time + η_ij_ × (treat x wheat) + μ_ijl_ × (treat × wheat × time) + ε_ijkl_, where α is the meaning effect; β, γ, π, η and μ are the adjusted coefficients of the fixed effects in the model; δ is the adjusted coefficient of the random effects in the model; ε is the random error associated with the *j*th treatment in experiment *k* assigned to the *i*th wheat at time l; subscripts *i, j, k* and *l* are the df of each factor: two wheat cultivars, three treatments, two experiments and five times. Time was considered as a repeated factor of the model. Regarding the effects of enzyme preparation on each cultivar, as the interaction ‘treatment × wheat × cumulative time’ was significant, a SLICE option was added to the LSMEANS statement in order to compute the treatment effect at each time for both cultivars separately. All statistical analyses were considered to be significant at *P* < 0.05.

## Results

### Carbohydrate compositions of different wheat cultivars

Sugar composition of the whole grain (Table 1) and the soluble extracts (Table 2) were determined for Caphorn, Tapidor, Apache, Oratorio, Aztec and Isengrain wheat cultivars. The overall monosaccharide content in the whole grains was found to be globally similar for the six cultivars (between 78.10% and 83.15%) even if Caphorn and Isengrain present the higher amounts of total monosaccharide (83.15% and 82.72%, respectively). Moreover, the Tapidor, Apache, Oratorio and Aztec whole grains were composed of a very close rate of arabinose, xylose and glucose (Table 1). Mannose and galactose were present at low levels with little variations between the six cultivars and therefore were not discussed further. The proportions of polysaccharides in the soluble extracts (Table 2) were however different between the six cultivars, with the highest levels of monosaccharides in the Caphorn and Isengrain extracts (9.95% and 4.09%, respectively). This was connected to high levels of glucose and total monosaccharide in the whole grain of both wheat cultivars. Furthermore, the Caphorn and Isengrain water extracts have shown the highest and the lowest AX contents (1.37% *vs* 0.54%) simultaneously with the lowest and the highest A:X ratio (0.68 *vs* 0.86). Finally, regarding these monosaccharide compositions, the AX amounts and the A:X ratio, Caphorn and Isengrain wheat cultivars were selected for the rest of the study for their contrasted soluble AX content and structure.

**Table 1:** **Carbohydrate composition into the whole grain of different wheat cultivars**

**Cultivar**	**Ara**	**Xyl**	**Man**	**Gal**	**Glc**	**Total**	**AX**
**Caphorn**	2.80	4.84	0.37	0.66	74.49	83.15	7.64
**Tapidor**	2.69	4.57	0.41	0.55	71.98	80.19	7.26
**Apache**	2.46	4.55	0.37	0.54	72.40	80.32	7.01
**Oratorio**	2.92	4.66	0.44	0.62	71.34	79.99	7.58
**Aztec**	2.62	4.79	0.44	0.58	69.67	78.10	7.41
**Isengrain**	2.40	4.00	0.49	0.56	75.27	82.72	6.40

Values are expressed in g/100 g of dry mass. Arabinose (Ara), xylose (Xyl), mannose (Man), galactose (Gal), glucose (Glc), arabinoxylan (AX).

**Table 2:** **Carbohydrate composition in the soluble extract from different wheat cultivars**

**Cultivar**	**Ara**	**Xyl**	**Man**	**Gal**	**Glc**	**Total**	**AX**	**A:X**
**Caphorn**	0.58	0.79	0.16	0.26	8.17	9.95	1.37	0.68
**Tapidor**	0.43	0.61	0.02	0.09	1.50	2.65	1.04	0.71
**Apache**	0.30	0.37	0.02	0.15	0.61	1.46	0.67	0.82
**Oratorio**	0.36	0.46	0.02	0.16	0.60	1.59	0.81	0.78
**Aztec**	0.34	0.43	0.03	0.16	0.69	1.65	0.77	0.79
**Isengrain**	0.26	0.28	0.18	0.21	3.15	4.09	0.54	0.86

Soluble extracts were prepared from 1 g of ground wheat grain in 4 mL of water [31]. Arabinose (Ara), xylose (Xyl), mannose (Man), galactose (Gal), glucose (Glc), arabinoxylan (AX), arabinose:xylose molar ratio (A:X). The results were expressed in g of component in water soluble extract/100 g of whole grain.

### Carbohydrate distribution into the different TIM-1 fractions after 360 minutes of digestion

In order to evaluate the carbohydrates distribution into the soluble fractions from the different compartments of the TIM-1 throughout the digestion time, the amount of glucose, xylose and arabinose was measured without xylanase supplementations (Table 3). Both wheat cultivars presented a similar carbohydrate global repartition although the 3 main monosaccharides were differently distributed: 80% and 81% of glucose were found into the dialysates of Caphorn and Isengrain, respectively, while only 11% and 14% of arabinose plus xylose were found in the same compartments. Conversely, arabinose and xylose were mainly distributed in the soluble fractions of ileal deliveries and jejuno-ileal residues. Overall, no differences were observed for arabinose and xylose contents in the jejunal and ileal dialysates from Caphorn and Isengrain cultivars. With regard to the ileal deliveries and the soluble fractions of the gastro-duodenal residues, much higher levels of arabinose (+37.5 mg; *P* = 0.018 and + 12.0 mg; *P* = 0.020, respectively) and xylose (+65.7 mg; *P* = 0.009 and + 20.2 mg; *P* = 0.006, respectively) were measured for Caphorn than for Isengrain. The glucose content did not present any significant difference in either wheat cultivars regardless of the collected fractions in the TIM-1. These results indicated that AX were not degraded in the absence of xylanase along the digestive tract and were in accordance with the soluble monosaccharide compositions of the cultivars (Table 2) where the AX content is higher in Caphorn than in Isengrain.

**Table 3:** **Distribution of soluble carbohydrates from ground wheat of Isengrain and Caphorn cultivars in the TIM-1 compartments**

**Wheat effect**		**Jejunal dialysis**	**SEM**	**Ileal dialysis**	**SEM**	**Ileal deliveries**	**SEM**	**Gastro-duodenal soluble residues**	**SEM**	**Jejuno-ileal soluble residues**	**SEM**	**Total, mg**	**SEM**	**Initial, mg**	**Total, %**
**Ara**	Caphorn	25.6	5.7	12.9	3.5	104.3	17.1	34.9	5.5	91.4	33.9	269.1	65.9	1,260	21.3
	Isengrain	22.5	0.02	12.1	1.5	66.8	14.6	22.9	1.3	71.0	5.7	195.3	23.2	1,080	18.0
**Xyl**	Caphorn	22.8	4.0	12.8	3.2	160.1	25.9	46.5	5.9	134.6	45.2	376.8	84.2	2,178	17.3
	Isengrain	17.3	0.4	9.3	0.05	94.4	23.6	26.3	0.6	91.5	8.7	238.8	33.4	1,800	13.3
**Glc**	Caphorn	12,605.6	215.4	5,975.0	778.0	1,776.3	1.5	1,403.1	299.7	1,437.8	439.4	23,197.8	1734.2	33,520	69.2
	Isengrain	13,992.2	53.1	5,519.9	159.4	1,914.7	416.4	985.5	57.6	1,553.0	22.4	23,965.3	390.2	33,871	70.7

The distribution (mg) of soluble arabinose (Ara), xylose (Xyl) and glucose (Glc) from 45 g (dry basis) of ground wheat, without xylanase supplementation, has been determined by gaz chromatography. The total expressed in percentage is calculated based on the initial content in mg. Mean values were based on three repetitions. SEM corresponds to the standard error of the mean estimated by the standard deviation (SD) divided by the square root of the sample size (n = 2), assuming statistical independence of the values in the sample.

### Total reducing ends as a global digestibility marker of wheat in the TIM-1 compartments – effects of xylanase supplementation

**Figure 2:**
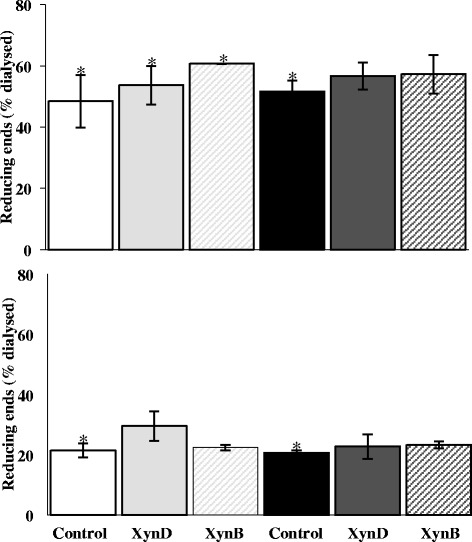
**Cumulative reducing ends in the dialysates with and without enzymatic treatment.** The cumulative reducing ends in the jejunal (upper) and ileal (lower) dialysates on Caphorn (left) and Isengrain (right) without xylanase (white and dark) with XynD (full light grey and full dark grey) and with XynB (hatched light grey and hatched dark grey) were expressed as a percentage of the total reducing ends measured after the 360 min of digestion in all the TIM-1 compartments. *Corresponds to the significant differences.

The total reducing ends were measured, both with and without xylanases added in TIM-1, in order to evaluate the extent of polysaccharide hydrolysis (Figure 2). As the reducing ends reflect both the hydrolysis of starch as well as NSP, these values can be considered as a marker of wheat digestion in the TIM-1. For both wheat cultivars, the total reducing ends in the jejunal and ileal dialysates represented the major part (70-80%) of the reducing ends measured in the TIM-1 after 360 min. Conversely, in the ileal deliveries and gastro-intestinal residues the total reducing ends represented only ~10% in each (data not shown). In accordance with the results presented above, total reducing ends dialyzing at the jejunal and ileal levels in the absence of xylanase (control trial) were in the same range for both cultivars, with 48.4 ± 8.6% and 21.4 ± 2.3% for Caphorn *vs* 51.7 ± 3.5% and 20.7 ± 0.9% for Isengrain, respectively. The XynD and XynB supplementations increased total reducing ends for both wheat cultivars although the effect was slightly lower on Isengrain. However for Caphorn, XynD was effective all along the digestion tract (+5.3% and +8.1% relative to the control in the jejunal and ileal dialysates, respectively), whereas XynB was mostly effective at the beginning of the digestion (+12.3% and +1.0% *vs* the control in the jejunal and ileal dialysates, respectively). Conversely, XynD and XynB had a comparable effect on Isengrain with +5.0% and +5.6% in the jejunal dialysate and +2.1% and +2.6% in the ileal dialysate, respectively. Finally, these results suggested that the hydrolysis performed by XynD and XynB in the presence of salivary/pancreatic α(1,4)-amylases occurred mainly in the proximal parts (from the gastric to the jejunal compartments) of the TIM-1.

### Kinetic effects on reducing ends release by the XynD and XynB supplementations on wheat digestion

**Figure 3:**
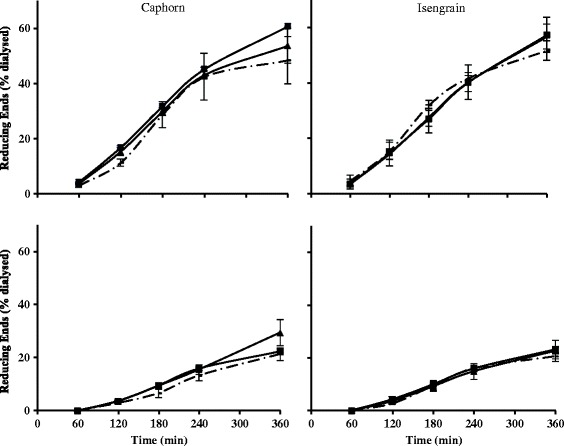
**Cumulative time courses of soluble reducing ends with and without enzymatic treatment.** The cumulative time courses of reducing ends in the jejunal (upper) and ileal (lower) dialysis from TIM-1 compartments on Caphorn (left) and Isengrain (right) wheat cultivars without xylanase (dashed line), with (full line) XynD (▲) and with XynB (■) were expressed in percentage of the total reducing ends dialyzed in the same compartment during the 360 min of digestion.

In order to follow the kinetic effect of a xylanase supplementation, the cumulative time course of reducing ends on the jejunal and the ileal dialysates is shown for XynD and XynB supplementation (Figure 3). For Caphorn cultivar, the XynB and XynD positive effect observed on Figure 2 appeared mainly at the end of the trials between 4 and 6 h. Indeed, the values of reducing ends measured at the previous times are not significantly different for the un-supplemented assay. However, the time course confirms that XynB was the most active at the jejunal level, whereas XynD was similarly active at both levels. When Isengrain was used, both enzymes tended to increase the contents in reducing ends in the jejunal dialysates during the last hours and gave a time course very similar to the control assay in the ileal dialysates.

### Effect of XynD and XynB supplementations on monosaccharide distribution on the TIM-1 compartments

**Figure 4:**
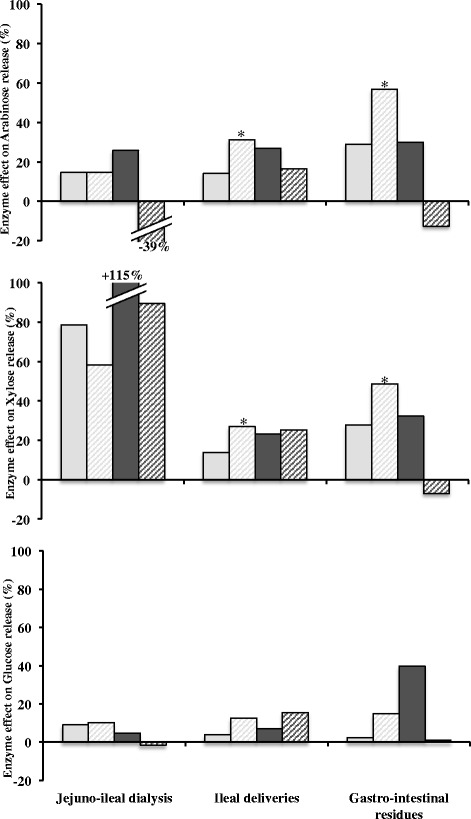
**XynD and XynB supplementation effects on Caphorn and Isengrain cultivars in the TIM-1 compartments.** The xylanase supplementation effects on Caphorn and Isengrain with XynD (full light grey and full dark grey) and with XynB (hatched light grey and hatched dark grey) were expressed in percentage of soluble arabinose (upper), xylose (medium) and glucose (lower) increase or decrease compared to the control without enzyme addition. *Corresponds to the significant difference.

In order to get an overview of the effect of xylanase supplementation on monosaccharide content for each of the wheat cultivars, the values obtained for each monosaccharide in each compartment have been pooled together to define both the gastro-intestinal residues, and the jejuno-ileal dialysates (Figure 4). The enzyme effect was expressed as the percent increase or decrease of the monosaccharide release compared to the control without enzyme. XynD and XynB additions significantly affected the contents in monosaccharide in all the samples collected after 360 min of digestion. XynD increased the arabinose and xylose contents in the different fractions and for both wheat cultivars. Specifically, XynD supplementation increased the arabinose amount for Caphorn and Isengrain with +14.5% and +25.6% into the jejuno-ileal dialysates, +14.1% and +26.8% into the ileal deliveries and +28.9% and +30.0% into the gastro-intestinal residues, respectively. The xylose content in the presence of XynD followed the increase of the arabinose amount with for Caphorn and Isengrain, +78.7% and +115.0% into the jejuno-ileal dialysates, +13.9% and +23.2% into the ileal deliveries and +27.7% and +32.3% into the gastro-intestinal residues, respectively. Finally, there was a trend for an increase of the glucose content for both wheat cultivars in the TIM-1 fractions collected in the presence of XynD. The largest increase of glucose content was observed on Isengrain cultivar into the jejuno-ileal dialysate (+39.7%).

The XynB effect on the arabinose release was close to the XynD effect for the Caphorn cultivar, with +14.7%, +31.3% and +56.8% into the jejuno-ileal dialysates, the ileal deliveries and the gastro-intestinal residues respectively. For Isengrain the effect was controversial with −39.0%, +16.4% and −12.6% for the three compartments, respectively. The XynB effect on xylose contents for Caphorn and Isengrain was +58.3% and +89.5% into the jejuno-ileal dialysates, +27.1% and +25.2% into the ileal deliveries and +48.6% and −6.9% into the gastro-intestinal residues, respectively. Furthermore, it is interesting to note that the XynB effect on glucose appears later in the digestive tract for Isengrain than for Caphorn, with an increase of +15.5% in the corresponding ileal delivery.

### Kinetics of glucose appearance in jejuno-ileal dialysis in TIM-1

**Figure 5:**
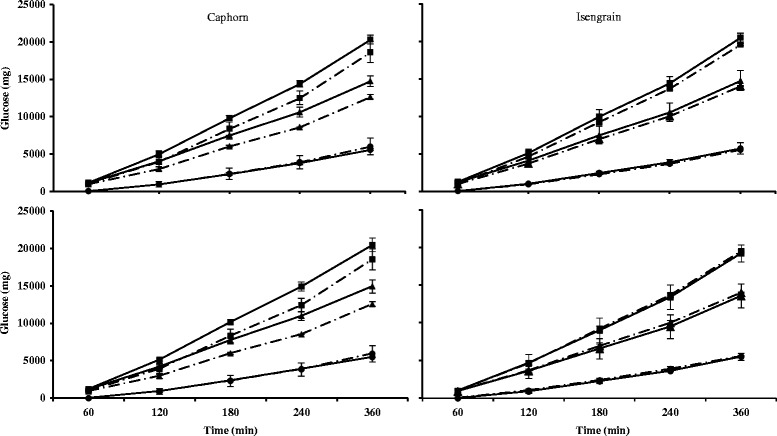
**Cumulative time courses of glucose from Caphorn and Isengrain wheat cultivars after xylanase treatments.** The cumulative time courses of glucose from Caphorn (left) and Isengrain (right) without (dashed line) xylanases or with (full line) XynD (upper) or XynB (lower) in the jejunal (▲), ileal (●) and jejuno-ileal (■) dialysis from TIM-1 digestible fractions were expressed in mg.

The kinetics of glucose appearance in dialysates was monitored in presence or absence of XynD and XynB in order to evaluate the impact of xylanase supplementation on the energy available for the animal (Figure 5). For Caphorn and Isengrain cultivars, the glucose dialysis mainly took place into the jejunal compartment (between 2.11 and 2.53 times higher than into the ileal compartment for Caphorn and Isengrain, respectively). In the jejunal dialysate at 360 min, the content of glucose was higher for Isengrain than for Caphorn (+1,386 mg; *P* = 0.021). There were no significant differences between the two wheat cultivars in the ileal dialysates regardless of either digestion time or enzymatic treatment. However, XynD and XynB significantly increased the glucose content into the jejunal dialysates and consequently into the total dialysates for Caphorn cultivar throughout the digestion time. Finally at 360 min, the XynD supplementation on Caphorn cultivar increased the jejunal and total glucose contents by 17% (*P* = 0.014) and 9% (*P* = 0.060), respectively. The XynB supplementation increased the glucose contents in the same fractions by 19% (*P* = 0.006) and 10% (*P* = 0.035), respectively.

The kinetics of appearance of arabinose and xylose in jejunal and ileal dialysates were almost identical to that of glucose, with the most evident effect of the enzymes during the last hours of transit. However, it should be noted that XynB showed a strong effect on xylose solubilisation from Isengrain cultivar in the jejunal compartment and no effect in the ileal dialysate confirming a proximal effect of XynB on AX hydrolysis (data not shown).

### Kinetics of arabinose, xylose and glucose appearance into the ileal delivery compartment

**Figure 6:**
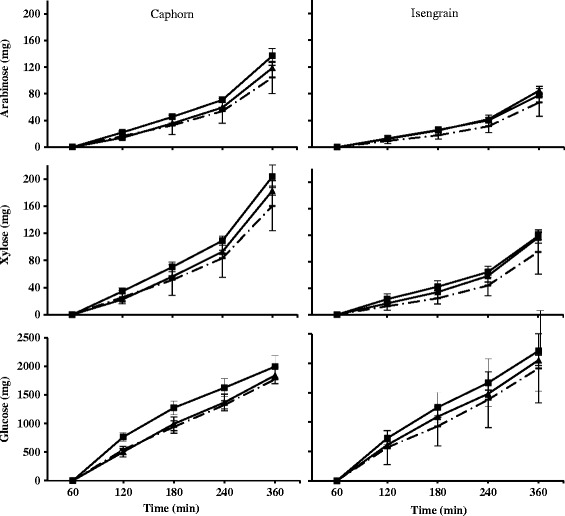
**Cumulative time courses of carbohydrates with and without xylanases supplementation in the ileal deliveries.** The cumulative time courses of arabinose (upper), xylose (medium) and glucose (lower) from Caphorn (left) and Isengrain (right) wheat cultivars without (dashed lines) xylanases supplementation and with (full line) XynD (▲) and XynB (■) in the soluble fraction of the ileal deliveries were expressed in mg.

The level of arabinose, xylose and glucose into the ileal deliveries was monitored along the digestion in order to evaluate the effect of xylanase supplementation on the monosaccharides and short oligosaccharides released which consequently transit to the colon compartment where they could play a prebiotic role (Figure 6) [33]. Without the enzyme, ileal deliveries recovered from Caphorn feeding presented higher arabinose content than Isengrain all along the time course (8.3% *vs* 6.2% at 360 min, *P* = 0.005), and xylose contents (7.4% *vs* 5.2% at 360 min, *P* = 0.003). No significant differences were observed for glucose contents, with 5.3% for Caphorn *vs* 5.7% for Isengrain, at 360 min (*P* = 0.664). The XynD supplementation increased arabinose (at 360 min, +13.8% (*P* = 0.149) in Caphorn and +26.9% (*P* = 0.089) in Isengrain), xylose (at 360 min, +14.4% (*P* = 0.172) in Caphorn and +23.4% (*P* = 0.178) in Isengrain) and glucose (at 360 min, +3.8% (*P* = 0.833) in Caphorn and +7.1% (*P* = 0.669) in Isengrain) contents in the soluble fraction of ileal deliveries. Conversely, XynB showed globally a stronger effect than XynD on Caphorn cultivar with +31.7% (*P* = 0.009) for arabinose, +26.9% (*P* = 0.022) for xylose and +12.5% (*P* = 0.493) for glucose contents. With Isengrain the increases were +16.4% (*P* = 0.264), +27.0% (*P* = 0.148) and +15.5% (*P* = 0.367), for the 3 sugars, respectively. Finally, it is noteworthy that the effect of xylanase supplementation mainly took place during the three first hours of digestion especially concerning the glucose amount compared to arabinose and xylose. Indeed, the amount of arabinose and xylose increased progressively all along the digestion time whereas the glucose amount quickly increased in the two first hours, before leveling off. Moreover, Caphorn cultivar was more sensitive to XynB than to XynD supplementation, whereas Isengrain cultivar was less sensitive than Caphorn to xylanase supplementation, regardless of the xylanases used.

## Discussion

In this study XynB (GH11) and XynD (GH10) from *T. versatilis* were evaluated for their ability to improve the digestibility of Caphorn and Isengrain wheat cultivars by using the *in vitro* gastro-intestinal model TIM-1. The sugar composition and AX ramification of both wheat cultivars studied here are in accordance with previous studies. Pritchard *et al.* determined the monosaccharide composition and the total A:X ratio of 211 wheat varieties and found that the total AX content ranges from 2.37% to 10.75% dry matter whereas the A:X ratio ranges from 0.4 to 1.3 [34]. Compared to Isengrain (0.54%) or to the literature values, the level of soluble AX measured in Caphorn was high (1.37%). Indeed, previous studies showed that soluble AX content in wheat whole grain ranges from 0.24% to 0.75% [35,36]. The viscosity of a polymer solution is dependent on different parameters: the primary structure of the polymer, its molecular weight and its concentration. Although AX structure and molecular weight can vary according to cultivars and environment, the amount of water-soluble AX is a good indicator of viscosity. In this study, Caphorn was found to contain water-soluble AX at three times higher levels than for Isengrain, which supports that Caphorn is a more viscous cultivar than Isengrain. As a matter of fact, previous authors reported that viscosity measurements, varied from 3.35 to 4.98 for Isengrain and from 7.86 to 10.2 for Caphorn [27-29].

Studies have demonstrated that chyme viscosity decreased by partial or complete hydrolysis of soluble NSP, supplementation in CAZymes (*e.g.* xylanases or β-glucanases) represents a good treatment to improve animal performance [7]. Based on this fact and previous work on xylanase supplementations, the preliminary step of this study required characterizing the behavior of both wheat cultivars along the *in vitro* gastro-intestinal model TIM-1. After 6 h digestion, it has been shown that the glucose levels in the dialysates were much higher than those of arabinose and xylose. This can be explained by a highest hydrolysis of glucose-containing polysaccharides (*i.e.* starch), compared to NSP (*e.g.* AX), releasing xylose and arabinose residues. In the TIM-1 model, the digestion process mimics the monogastric digestion, where only polysaccharides such as starch can be digested by salivary, gastric and intestinal enzymes. The soluble xylose and arabinose found in the different TIM-1 fractions provided from the hydrolysis of AX, originally located into the plant cell wall [37]. The gastro-duodenal residues and the ileal deliveries representative of the undigested fractions, present some differences in the soluble fractions with much more arabinose and xylose for Caphorn than for Isengrain. This difference is mainly linked to the fact that both wheat cultivars have different initial soluble polysaccharides contents, with especially a higher level of AX in Caphorn.

The monitoring of time courses for sugar appearances in dialysates showed that the glucose dialysis mainly took place in the jejunal compartment. This observation was in accordance with the fact that α(1,4)-amylase and gluco-amylase quantities and activities were higher in this proximal part of the intestinal tract than in the distal parts [38]. In addition, there was slightly more dialyzed glucose for Isengrain than for Caphorn, suggesting that the starch digestion was more efficient in this cultivar. These results were in agreement with those obtained previously by Preynat *et al.* and Lafond *et al.* [39,25].

Hübener *et al.* and Torok *et al.* demonstrated that the CAZymes supplementation improves animal performance and nutrient utilization and also changes the composition and metabolic potential of bacterial populations in the gut microbiota [40,41]. Taking those different beneficial effects in consideration, the present work allowed estimation of the effects of XynD (GH10) and XynB (GH11) supplementations on Caphorn and Isengrain wheat cultivars. The addition of these enzymes in the TIM-1 induced an increase of the levels of xylose, arabinose and glucose into the dialysates fractions (corresponding to the physiological intestinal absorption), suggesting that the AX hydrolysis favored the starch degradation. In this regard, Classen *et al.* suggested that wheat NSP might act as a physical barrier preventing or slowing access of digestive enzymes to starch granules [42]. Romero *et al.* confirmed these observations in showing that a blend of β(1,4)-xylanase, α(1,4)-amylase and protease improved ileal digestibility of starch, fat and protein and thus significantly improved the poultry’s ability to extract energy [43]. The microscopy study of Bedford and Autio reinforces the fact that there was indeed a considerable amount of starch surrounded by intact cell walls in the intestinal digesta of broilers fed wheat-based diets, which was mostly removed after NSP-degrading enzyme supplementation [5]. This hypothesis is strengthened by XynD and XynB supplementations that increased reducing ends content, with a direct effect on the soluble arabinose and xylose release and an indirect effect on glucose release (in both case mono- and/or oligosaccharides were produced), in the different TIM-1 collected fractions. Xylanase addition contributed to partial or complete degradation of soluble AX into arabinoxylooligosaccharides (AXOS) that were found primarily in the proximal part of the gut (*i.e.* jejunal dialysates) and also in a weak amount in the ileal deliveries. Consequently, the high amount of AXOS released from AX by both enzymes could: promotes a prebiotic effect on the colic microbiota, modulates the intestinal microbiota, and reduce the pathogens population in the gut and prevent some intestinal diseases [44,45].

Accordingly, Aulrich and Flachowsky found that the *in vitro* supplementation of the combination β(1,4)-xylanase plus β(1,4)-glucanase in wheat-based diet increased the amount of arabinose and xylose and reduced AX amount suggesting that solubilisation of insoluble cell wall polysaccharides by enzymatic hydrolysis and the degradation of solubilized material occurred simultaneously [46]. Isengrain cultivar showed a quite higher A:X ratio than Caphorn suggesting that xylan backbone of Isengrain AX is sensibly more substituted by arabinose than that of Caphorn AX. In our study, XynB had a greater global effect on Caphorn AX suggesting that it was much more efficient on low-substituted AX, whereas XynD had a greater global effect than its homologous from family GH11 on Isengrain which exhibits a higher substitution degree. These results are consistent with previous reports, where we observed that the XynD from *T. versatilis* was able to cleave the highly substituted xylan whereas XynB preferred less substituted xylan [19,20]. Moreover, it is known that xylanases from GH10 family are able to cleave the AX backbone in decorated regions and are less hampered by the presence of arabinose substitutions along the xylan backbone than GH11 members [47]. Although our experiments were carried out in a very complex system on solid matter and in non-optimal conditions for the enzymes, our results are directly linked to what was previously demonstrated in simple soluble media, containing only one substrate and one enzyme in optimal conditions. Consequently, it may be conceivable to observe a synergetic effect with supplementation of the two xylanases together. This assumption is based on the fact that XynD is active throughout the digestive tract while XynB shown a strong activity on the proximal part of the gut. These results were correlated to the membership of two different CAZy families and thus suggest a different mode of action *in vivo*.

In this study we have shown globally a proximal effect of both enzymes, which decreased with digestion time. As we already described in 2010 for the Rovabio Excel^TM^ the reduction in activity might be due either to enzyme degradation by the proteases available in TIM-1 or to a limitation in substrates [25]. However, based on previous studies [19,48], the recombinants XynB and XynD are both highly N- and O-glycosylated and it is well known that these kind of post-translational modifications induce a relative resistance to the protease [49]. We can thus suggest that N- and O-glycosylations of both enzymes confer a proteolysis resistance during the first times of digestion followed by a progressive degradation, inducing a loss of xylanolytic activity.

Finally, XynD and XynB were both sensitive to the wheat proteinaceous inhibitors XIP-I (Xylanase-Inhibitor-Protein-I) with a *K*_i_ of 10.2 and 89.7 nmol/L, respectively, whereas only XynB was found to be sensitive to TAXI (*Triticum aestivum* Xylanase Inhibitor) with a *K*_i_ of 2.9 nmol/L (unpublished data from Lafond, [48,50]). Based on literature, Isengrain whole grain contains 560 μg/g of XIP-I and 190 μg/g of TAXI [51], which leads one to consider if these inhibitory levels are higher in Caphorn, and suggest a reduction of enzyme activities.

## Conclusions

In the present study, the TIM-1 fed with Caphorn or Isengrain-based diets supplemented into endo-β(1,4)-xylanases D (GH10) or B (GH11) from *Talaromyces versatilis* have shown a significant increase into glucose, arabinose and xylose contents in the jejunal dialysates and into arabinose and xylose contents in ileal deliveries. Otherwise, the results showed that XynD acted throughout the digestive tract while XynB was mainly active on the proximal part of the gut, suggesting a different mode of action of the two enzymes. Together, these data suggest that XynD and XynB supplementation in wheat-based diet could alleviate the anti-nutritional effects of NSP (*e.g.* AX) and consequently could enhance the access of amylases to starch by limiting the physical entrapment of starch. In this manner, XynD and XynB would promote the soluble glucose intestinal absorption, which constitutes the highest metabolizable energy. Finally, due to the versatility and the different modes of action of the two enzymes, it would be interesting to manage a supplemental study in order to confirm the XynD and/or XynB efficiencies used alone or in combination with other CAZymes into *in vivo* trials.

## Abbreviations

Ara: Arabinose
AX: Arabinoxylan
A:X: AX with different ratio Arabinose/Xylose
AXOS: Arabinoxylooligosaccharides
CAZyme: Carbohydrate active enzyme
DNS: Dinitrosalicylic acid
Gal: Galactose
GH: Glycoside hydrolase
GLC: Gaz liquid chromatography
Glu: Glucose
ICBN: International code of botanical nomenclature
Man: Mannose
NSP: Non starch polysaccharides
TIM-1: TNO gastrointestinal model-1
XIP-I: Xylanase-Inhibitor-Protein-I
Xyl: Xylose
XynB: Endo-β(1,4)-xylanase B
XynD: Endo-β(1,4)-xylanase D

## References

[1] Austin SC, Wiseman J, Chesson A (1999). Influence of non-starch polysaccharides structure and the metabolisable energy of U.K. wheat fed to poultry. J Cereal Sci.

[2] Gutiérrez-Alamo A, Pérez de Ayala P, Verstegen MWA, Den Hartog LA, Villamide MJ (2008). Variability in wheat: factors affecting its nutritional value. World’s Poult Sci J.

[3] Slominski BA, Gdala J, Boros D, Campbell LD, Guenter W, Jones O. Variability in chemical and nutritive composition of Canadian wheat and the potential for its minimization by enzyme use. In: Proceeding of the XXI World Poultry Congress, Montreal, Canada; 2000 (CD-ROM)

[4] Thender O, Westerlund E, Aman P, Graham H (1989). Plant cell walls and monogastric diets. Anim Feed Sci Technol.

[5] Bedford MR, Autio K (1996). Microscopic examination of feed and digesta from wheat-fed broiler chickens and its relation to bird performance. Poult Sci.

[6] Wiseman J, Nicol NT, Norton G (2000). Relationship between apparent metabolisable energy (AME) values and *in vivo*/ *in vitro* starch digestibility of wheat for broilers. World Poult Sci J.

[7] Kiarie E, Romero LF, Nyachoti CM (2013). The role of added feed enzymes in promoting gut health in swine and poultry. Nutr Res Rev.

[8] Henry RJ (1987). Pentosan and (1–3)(1–4)-β-glucan concentrations in endosperm and wholegrain of wheat, barley, oats and rye. J Cereal Sci.

[9] Choct M: Feed non-starch polysaccharides: chemical structures and nutritional significance. In: Feed Milling International 1997, 13–26.

[10] Saulnier L, Guillon F, Chateigner-Boutin AL (2012). Cell wall deposition and metabolism in wheat grain. J Cereal Sci.

[11] Bedford MR, Classen HL (1993). An *in vitro* assay for prediction of broiler intestinal viscosity and growth when fed rye-based diets in the presence of exogenous enzymes. Poult Sci.

[12] Mathlouthi N, Mallet S, Saulnier L, Quemener B, Larbier M (2002). Effect of xylanase and β-glucanase addition on performance, nutrient digestibility, and physico-chemical conditions in the small intestine contents and caecal microflora of broiler chickens fed a wheat and barley-based diet. Anim Res.

[13] Meng X, Slominski BA, Nyachoti CM, Campbell LD, Guenter W (2005). Degradation of cell wall polysaccharides by combinations of carbohydrase enzymes and their effect on nutrient utilization and broiler chicken performance. Poult Sci.

[14] Maisonnier-Grenier S, Clavurier K, Saulnier L, Bonnin E, Geraert PA (2006). Biochemical characteristics of wheat and their relation with apparent metabolisable energy value in broilers with or without non-starch polysaccharide enzyme. J Sci Food Agric.

[15] Norvell LL (2011). Fungal nomenclature. 1. Melbourne approves a new Code. *Mycotaxon*. Mycotaxon.

[16] Samson RA, Yilmaz N, Houbraken J, Spierenburg H, Seifert KA, Peterson SW (2011). Phylogeny and nomenclature of the genus *Talaromyces* and taxa accommodated in *Penicillium* subgenus *Biverticillium*. Stud Mycol.

[17] Cantarel BL, Coutinho PM, Rancurel C, Bernard T, Lombard V, Henrissat B (2009). The Carbohydrate-Active EnZymes database (CAZy): an expert resource for Glycogenomics. Nucleic Acids Res.

[18] Guais O, Borderies G, Pichereaux C, Maestracci M, Neugnot V, Rossignol M (2008). Proteomics analysis of “Rovabio Excel”, a secreted protein cocktail from the filamentous fungus *Penicillium funiculosum* grown under industrial process fermentation. J Ind Microbiol Biotechnol.

[19] Lafond M, Tauzin A, Desseaux V, Bonnin E, Ajandouz EH, Giardina T (2011). GH10 xylanase D from *Penicillium funiculosum*: biochemical studies and xylooligosaccharide production. Microbial Cell Fact.

[20] Lafond M, Guais O, Maestracci M, Bonnin E, Giardina T: Four GH11 xylanases from the xylanolytic fungus *Talaromyces versatilis* act differently on (arabino)xylans. Appl Microb Biotechnol 2014, In press. 10.1007/s00253-014-5606-x 24664446

[21] Minekus M, Marteau P, Havenaar R, Huis In’t Veld JHJ (1995). A multicompartmental dynamic computer-controlled model simulating the stomach and small-intestine. ATLA.

[22] Minekus M, Jelier M, Xiao J, Z Kondo S, Iwatsuki K, Kokubo S (2005). Effect of partially hydrolyzed guar gum (PHGG) on the bioaccessibility of fat and cholesterol. Biosci Biotechnol Biochem.

[23] Haraldsson AK, Rimsten L, Alminger M, Andersson R, Åman P, Sandberg AS (2005). Digestion of barley malt porridges in a gastrointestinal model: Iron dialysability, iron uptake by Caco-2 cells and degradation of [beta]-glucan. J Cereal Sci.

[24] Fassler C, Arrigoni E, Venema K, Hafner V, Brouns F, Amado R (2006). Digestibility of resistant starch containing preparations using two *in vitro* models. Eur J Nutr.

[25] Lafond M, Bouza B, Eyrichine S, Bonnin E, Crost EH, Geraert PA (2011). An integrative *in vitro* approach to analyse digestion of wheat polysaccharides and the effect of enzyme supplementation. Br J Nutr.

[26] Choct M, Annison G (1992). The inhibition of nutrient digestion by wheat pentosans. Br J Nutr.

[27] del Alamo Gutiérrez A, Verstegen MWA, DenHartog LA, Pérez de Ayala P, Villamide MJ (2008). Effect of wheat cultivar and enzyme addition to broiler chicken diets on nutrient digestibility, performance, and apparent metabolizable energy content. Poult Sci.

[28] Gutiérrez del Alamo A, Verstegen MWA, den Hartog LA, Pérez de Ayala P, Villamide MJ (2009). Wheat starch digestion rate affects broiler performance. Poult Sci.

[29] Gutiérrez del Alamo A, Pérez Ayala P, Den Hartog LA, Verstegen MWA, Villamide MJ (2009). Wheat starch digestion rate in broiler chickens is affected by cultivar but not by wheat crop nitrogen fertilization. Br Poult Sci.

[30] Bailey MJ, Biely P, Poutanen K (1992). Interlaboratory Testing of Methods for Assay of Xylanase Activity. J Biotechnol.

[31] Dervilly G, Saulnier L, Roger P, Thibault JF (2000). Isolation of homogeneous fractions from wheat water-soluble arabinoxylans. Influence of the structure on their macromolecular characteristics. J Agric Food Chem.

[32] Saulnier L, Peneau N, Thibault JF (1995). Variability in grain extract viscosity and water-soluble arabinoxylan content in wheat. J Cereal Sci.

[33] Childs CE, Röytiö H, Alhoniemi E, Fekete AA, Forssten SD, Hudjec N (2014). Xylo-oligosaccharides alone or in synbiotic combination with Bifidobacterium animalis subsp. lactis induce bifidogenesis and modulate markers of immune function in healthy adults: a double-blind, placebo-controlled, randomised, factorial cross-over study. Br J Nutr.

[34] Pritchard JR, Lawrence GJ, Larroque O, Li Z, Laidlaw HKC, Morell MK (2011). A survey of β-glucan and arabinoxylan content in wheat. J Sci Food Agric.

[35] Finnie SM, Bettge AD, Morris CF (2006). Influence of cultivar and environment on water-soluble and water-insoluble arabinoxylans in soft wheat. Cereal Chem.

[36] Saulnier L, Sado PE, Branlard G, Charmet G, Guillon F (2007). Wheat arabinoxylans: exploiting variation in amount and composition to develop enhanced varieties. J Cereal Sci.

[37] Cosgrove DJ (2005). Growth of the plant cell wall. Nat Rev Mol Cell Biol.

[38] Kidder DE, Manners MJ (1980). The level of distribution of carbohydrases in the small intestine mucosa of pigs from 3 weeks of age to maturity. Br J Nutr.

[39] Preynat A, Gady C, Saulnier L, Bonnin E, Geraert PAG. Can we predict enzyme response in relation with wheat cultivar in broilers? In: 17th European Symposium on Poultry Nutrition, Edinburgh, Scotland, 2009 (Poster)

[40] Hübener K, Vahjen W, Simon O (2002). Bacterial responses to different dietary cereal types and xylanase supplementation in the intestine of broiler chicken. Arch Anim Nutr.

[41] Torok VA, Ophel-Keller K, Loo M, Hughes RJ (2008). Application of methods for identifying broiler chicken gut bacterial species linked with increased energy metabolism. Appl Environ Microbiol.

[42] Classen HL (1996). Cereal grain starch and exogenous enzymes in poultry diets. Anim Feed Sci Technol.

[43] Romero LF, Plumstead PW, Ravindran V (2011). Energy contribution of digestible starch, fat, and protein in response to combinations of exogenous xylanase, amylase, and protease in corn-based broiler diets. Poult Sci.

[44] Courtin CM, Broekaert WF, Swennen K, Lescroart O, Onagbesan O, Buyse J (2008). Dietary inclusion of wheat bran arabinoxylooligosaccharides induces beneficial nutritional effects in chickens. Cereal Chem.

[45] Nian F, Guo YM, Ru YJ, Péron A, Li FD (2011). Effect of xylanase supplementation on the net energy for production, performance and gut microflora of broilers fed corn/soy-based diet. Asian-Aust J Anim Sci.

[46] Aulrich K, Flachowsky G (1998). Studies on the mode of action of non-starch-polysaccharides (NSP) degrading enzymes *in vitro*. 1-Communication: Effects on the fractions of NSP. Arch Anim Nutr.

[47] Biely P, Vrsanskà M, Tenkanen M, Kluepfel D (1997). Endo-beta-1,4-xylanase families: differences in catalytic properties. J Biotechnol.

[48] Brutus A, Villard C, Durand A, Tahir T, Furniss C, Puigserver A (2004). The inhibition specificity of recombinant *Penicillium funiculosum* xylanase B towards wheat proteinaceous inhibitors. Biochim Biophys Acta.

[49] Pratap J, Rajamohan G, Dikshit K (2000). Characteristics of glycosylated streptokinase secreted from *Pichia pastoris*: enhanced resistance of SK to proteolysis by glycosylation. Appl Microbiol Biotechnol.

[50] Elliott GO, McLauchlan WR, Williamson G, Kroon P (2003). A wheat xylanase inhibitor protein (XIP-I) accumulates in the grain and has homologues in other cereals. J Cereal Sci.

[51] Bonnin E, Daviet S, Gebruers K, Delcour JA, Goldson J, Juge N (2005). Variation in the levels of the different xylanase inhibitors in grain and flour of 20 French wheat cultivars. J Cereal Sci.

